# Epidemiological trends and future burden of skin and subcutaneous diseases in China: insights from the global burden of disease study 2021

**DOI:** 10.3389/fmed.2026.1677296

**Published:** 2026-01-20

**Authors:** Zhongsong Zhang, Hang Su, Lifan Xiao, Chao Chang, Chengjie Wang, Heng Quan, Mao Lu

**Affiliations:** 1 Department of Dermatovenereology, Clinical Medical College and the First Affiliated Hospital of Chengdu Medical College, Chengdu, Sichuan, China; 2 School of Clinical Medicine, Chengdu Medical College, Chengdu, China; 3 School of Stomatology, Xinjiang Second Medical College, Karamay, China

**Keywords:** disease prediction, GBD, population aging, public health, skin and subcutaneous diseases

## Abstract

**Background:**

Skin and subcutaneous diseases (SSDs) constitute a major component of the non-fatal disease burden in China, with patterns that have evolved significantly over recent decades. Understanding long-term trends in incidence, prevalence, and mortality, as well as projecting future dynamics, is essential to inform public health strategies and support effective allocation of healthcare resources.

**Methods:**

We used data from the Global Burden of Disease Study 2021 (GBD 2021) to analyze the burden of SSDs in China from 1990 to 2021. We estimated incidence, prevalence, and mortality numbers alongside age-standardized rates, employing the Joinpoint model to calculate the annual average percentage change (AAPC) and age-period-cohort (APC) analyses for assessing trends in the burden of skin and subcutaneous diseases. Finally, ARIMA models were used to forecast age-standardized mortality rates through 2050.

**Results:**

From 1990 to 2021, the age-standardized incidence rate (ASIR) of SSDs in China exhibited a modest upward trend (AAPC = 0.11%), while the age-standardized prevalence rate (ASPR) increased more rapidly (AAPC = 0.29%). In contrast, the age-standardized mortality rate (ASMR) declined markedly over the same period (AAPC = −3.60%). In 2021, the ASIR reached 50,120 per 100,000 population (95% UI: 47,601–52,796), while the ASPR was 24,852 per 100,000 (95% UI: 24,126–25,562). Mortality remained low at 0.40 per 100,000 (95% UI: 0.33–0.48); however, YLL and YLD rates indicated a persistent disease burden, particularly among males, who exhibited higher YLLs than females. Joinpoint regression identified consistent period-related increases in incidence and prevalence alongside sustained declines in mortality risk. Age–period–cohort analysis further showed that mortality risk increased with age, whereas incidence and prevalence rose steadily across successive birth cohorts. Projections indicate that age-standardized mortality is likely to remain low and stable over the next decade.

**Conclusion:**

This study provides the first age- and sex-stratified projections of skin and subcutaneous disease burden in China through 2050, based on GBD 2021 data and age–period–cohort modeling. By identifying birth cohorts with the most rapidly increasing disability-adjusted life year (DALY) burden, these findings help identify priority populations and regions for targeted screening, guideline refinement, and pollution control interventions.

## Introduction

Skin and subcutaneous diseases (SSDs) include many conditions such as dermatitis, psoriasis, acne, and infections caused by fungi or bacteria (1, 2). Although rarely fatal, these conditions impose a substantial non-fatal disease burden and markedly reduce quality of life (3). Around the world, SSDs are among the top causes of years lived with disability (4). This shows their importance as a main source of ongoing health problems. Epidemiologically, SSDs affect individuals across all ages, with prevalence typically increasing in older populations due to cumulative exposures, age-related skin changes, and comorbidities that heighten vulnerability to infections and other dermatoses. Even though they are common and affect well-being and healthcare systems, SSDs have often received less research and policy attention than other long-term diseases (5–7). The rapidly changing population structure, accelerated urbanization, and recent changes in public health in China make it a key environment for studying the epidemiological trends and future burden of skin and subcutaneous diseases in China (8). Through longitudinal trend analysis and scenario-based forecasting, this study describes past and future disease burdens and evaluates how population aging, urbanization, and healthcare system reforms affect disease patterns and service demand. Given China’s vast population and rapid pace of change, our research findings provide important evidence to guide national health planning, resource allocation, and targeted prevention strategies (9, 10). In this study we adopt standard indicators from the Global Burden of Disease (GBD) framework. Specifically: “incidence” is the number of new SSDs cases occurring in a specified period; “prevalence” is the number of existing SSDs cases at a particular time (point prevalence) or over a period (period prevalence); the “mortality rate” is the frequency of deaths due to SSDs in a population over a defined time. We report disability-adjusted life years (DALYs), calculated as the sum of years of life lost (YLLs) from premature mortality and years lived with disability (YLDs) due to non-fatal health loss. Unless otherwise noted, indicators are presented as age-standardized rates per 100,000 population and/or absolute counts, consistent with GBD conventions.

The development of SSDs is influenced by a variety of mechanisms, including environmental, immunological, and social factors. In the context of urbanization and lifestyle changes in China, rapid industrialization, increased pollution, and altered dietary and living conditions are contributing to the rising incidence of SSDs (11). Environmental factors, such as exposure to air pollution and extreme weather, exacerbate conditions like dermatitis, psoriasis, and acne. Moreover, urban living, with its associated stressors and reduced green spaces, impacts immune function, leading to increased susceptibility to infections and skin disorders. Social factors, including changes in healthcare access and the increasing use of digital devices, also play a role in the growing burden of SSDs in China (12, 13). In addition, in China, rapid urban growth, changes in the age structure, and new lifestyles over the last 30 years have likely changed the patterns of SSDs burden (13). An aging population, environmental problems like pollution, and changing access to healthcare may lead to new trends in how often these diseases occur (14, 15). While global estimates show that there are many cases in China, there are not many detailed studies of these long-term trends and how they vary by age or other factors (16). Existing analyses using GBD 2017–2019 quantified crude SSD prevalence or a single subtype but (i) covered shorter time spans (< 30 y), (ii) omitted age-period-cohort (APC) disentanglement, and (iii) offered no data-driven forecasts. Recent GBD 2021 updates provided global overviews yet lacked China-specific deep dives. Consequently, policy makers still lack evidence on whether China’s SSDs trajectory differs from prior projections and which birth cohorts or periods drive change. Our study fills this gap by integrating APC modelling and ARIMA forecasting across the full 1990–2021 GBD 2021 series, thereby generating the first sex- and age-stratified 2050 projections for China. Addressing the above gap, our work provides three advances: (1) Methodological—we integrate APC decomposition with ARIMA forecasting to generate the first long-term (1990–2050) trajectories for six standard disease-burden metrics in China; (2) Granularity—we produce sex- and five-year-age-group-specific estimates, revealing a persistent male > female YLL differential unexplored in previous national reports; (3) Comparative insight—by benchmarking GBD 2021 results against earlier GBD 2017/2019-based studies, we uncover a slower-than-projected rise in DALYs after 2015, suggesting policy interventions may be yielding returns earlier than anticipate. Deaths from SSDs are low but can still happen in older people or those with weak immune systems, so it is important to watch how these numbers change over time (17, 18). Understanding how rates have changed over time and the effects of age, time period, and birth group is important for good public health planning. Joinpoint regression helps find when trends change, while age–period–cohort models separate effects due to aging, calendar time like policy changes, and differences between generations (19, 20). Similar modelling strategies have been used in other population-health settings: Joinpoint regression has been widely applied to identify inflection points in long-term incidence and mortality trends (e.g., cancers and major non-communicable diseases), and APC models have been used to disentangle cohort-driven transitions in cardiometabolic and respiratory conditions using national surveillance and GBD-derived time series (21–23). Also, using time-series forecasting can help predict future disease burden so that healthcare needs can be planned, resources allocated, and prevention strategies chosen. In particular, ARIMA and related time-series methods have been adopted to project mortality and DALY trajectories for conditions such as cardiovascular disease, stroke, diabetes, and selected cancers, supporting short- to mid-term forecasting for health-system planning (23–25). The findings of this study will provide crucial insights into China’s ongoing healthcare reform, particularly regarding the prevalence and future burden of skin and subcutaneous tissue diseases. We will propose practical and feasible policy recommendations based on this. At the same time, these recommendations aim to provide guidance for policy makers, public health officials, and healthcare professionals to more effectively address current public health challenges.

Building on previous GBD-based analyses, this study extends the temporal window to 2021, applies APC models and ARIMA forecasting, and directly contrasts our findings with earlier GBD 2019/2017 reports to provide a comprehensive view of China’s SSDs burden from 1990 to 2050, with an explicit aim of generating age- and locality-specific evidence for clinical screening schedules and Healthy China 2030 policy targets. We examine trends in incidence, prevalence, and deaths with Joinpoint regression, measure age, period, and cohort effects with simple models, and predict future trends up to 2050 using forecasting methods. Our goal is to help guide policy and highlight ways to address the growing burden of SSDs in China’s changing population. Especially in the context of rapid population aging, elucidating the impact of SSDs on public health systems and elderly health policies has become an urgent research topic.

## Materials and methods

### Data source

This study used data from the Global Burden of Disease Study 2021 (26), which provides systematic estimates of disease burden across countries and over time. The GBD database collects information from many sources, such as national health surveys, hospital records, cohort studies, and vital registration systems. This helps ensure broad coverage and reliable data. All original data for this study came from the publicly available GBD 2021 database.^1^ We extracted data for 1990 to 2021 on skin and subcutaneous diseases in China. For the data retrieval process, we utilized the GBD 2021 database’s search tool, applying specific disease categories related to skin and subcutaneous diseases, and including the relevant cause codes for these conditions, we define skin and subcutaneous diseases (SSDs) based on the classification system used by the GBD 2021 and the ICD-10. These diseases encompass a wide range of conditions, including dermatitis, psoriasis, acne, fungal infections, and subcutaneous infections. The full list of specific diseases categorized under SSDs, according to GBD 2021, includes: Dermatitis (e.g., Atopic dermatitis, Contact dermatitis, Seborrheic dermatitis), Psoriasis, Bacterial skin diseases (e.g., Cellulitis, Pyoderma), Scabies, Fungal skin diseases, Alopecia areata, Viral skin diseases, Acne vulgaris, Pruritus, Urticaria, Decubitus ulcer, other skin and subcutaneous diseases.

In China, several independent domestic data sources related to skin and subcutaneous diseases are available, including hospital-based medical record systems, regional epidemiological surveys, disease surveillance point (DSP) systems, and condition-specific studies focusing on selected SSD subtypes. However, these domestic datasets are typically limited in geographic coverage, time span, or disease scope, and often lack standardized age–sex stratification and unified burden metrics such as YLDs and DALYs. As a result, they are not well suited for long-term, nationwide trend analysis or future burden projection. Previous comparisons suggest that GBD estimates for China are generally consistent with domestic epidemiological evidence in terms of overall prevalence patterns and the predominance of non-fatal burden for SSDs, while providing improved temporal continuity and comparability through standardized modeling and data integration. Therefore, GBD 2021 was selected as the primary data source for this study to ensure national representativeness, methodological consistency, and suitability for trend analysis and forecasting.

The search criteria included data for all sex and age groups, and the relevant indicators, such as incidence, prevalence, mortality, YLLs, YLDs, and DALYs, incorporating 95% uncertainty intervals (UIs) (27, 28). This study is based on publicly available aggregated data from the Global Burden of Disease Study 2021 (26). Since the data used in this study does not involve personally identifiable information and is publicly available, ethical approval from an ethics committee or institutional review board was not required. Furthermore, informed consent was not necessary as no individual-level data was used in the analysis (26).

### Study design and population

This was a nationwide, population-based observational study examining trends in skin and subcutaneous disease burden in China from 1990 to 2021. The study population included all age and sex groups in mainland China as defined by GBD 2021. We extracted annual estimates of incidence, prevalence, mortality, YLLs, YLDs, and DALYs for these diseases using the publicly available GBD 2021 database. Age-standardized rates were calculated based on the GBD global standard population.

### Variable definition and justification

This study focuses on six standardized indicators directly provided by GBD -2021 and officially recommended by WHO and IHME to measure the burden of disease: ① Incidence rate (ASIR); ② Disease prevalence rate (ASPR); ③ Mortality rate (ASDR); ④ Disability Adjusted Life Years (DALYs); ⑤ Years of disability due to illness (YLDs); ⑥ Premature death years (YLLs). These indicators represent the burden of disease incidence, stock, mortality, and disability, and are the most internationally comparable set of core variables. In addition, studies on the burden of skin diseases published in the past decade have used this six-indicator combination for cross-country and cross period comparisons, demonstrating its ability to capture the main public health impacts of non-lethal diseases. This study uses the same variables to facilitate comparison with existing evidence and serve policy evaluation.

### Statistical analysis

We performed several steps to analyze the historical burden of SSDs in China using the six-outcome metrics justified in Section Variable definition and justification and to project future trends. Our work combined APC-plus-ARIMA framework, rarely applied to dermatologic burden research, enables forward-looking insights that move beyond descriptive retrospection. All analyses were performed using R software (version 4.4.0). Statistical significance was set at *p* < 0.05 where applicable.

### Descriptive analysis

We summarized absolute numbers and age-standardized rates of incidence, prevalence, mortality, YLLs, YLDs, and DALYs for 2021. Results were divided by sex and age group. We also used charts to show the distribution of cases and rates by age and sex.

### Joinpoint regression analysis

Joinpoint regression model is a collection of linear statistical models used to assess the trends in the burden of Skin and Subcutaneous Diseases over time. This model estimates the changes in morbidity rates using the least squares method, avoiding the subjectivity of typical trend analyses based on linear trends. The turning points in trends are determined by calculating the sum of squared residuals between estimated and actual values (29, 30). We assessed trends in age-standardized incidence, prevalence, and mortality rates from 1990 to 2021 using Joinpoint regression. Given the length of the study period, the maximum number of joinpoints was set to four, consistent with recommendations from the Joinpoint Regression Program for long-term population-level trend analyses. The final number of joinpoints was determined through a permutation test approach, which compares models with differing numbers of joinpoints and selects the most parsimonious model that provides a statistically significant improvement in fit.

Analyses were conducted assuming a log-linear model, in which annual rates were logarithmically transformed to stabilize variance and allow interpretation of results as constant percentage changes over time. A minimum of four data points was required between two joinpoints to ensure model stability and avoid overfitting. The Joinpoint Regression Program (Version 5.1.0, National Cancer Institute, USA) was used to identify significant changes in trend segments and to estimate the annual percent change (APC) for each segment, along with corresponding 95% confidence intervals (CIs). Statistical significance was assessed at a two-sided *α* level of 0.05. We also calculated the average annual percent change (AAPC) to summarize the overall trend across the entire study period, with AAPC computed as a weighted average of segment-specific APCs, where weights were proportional to the length of each time segment.

### Age–period–cohort analysis

The age–period–cohort (APC) model is a widely used analytical approach in epidemiology for disentangling the effects of age, period, and birth cohort on disease trends. By modeling rates as a function of age groups, calendar periods, and birth cohorts, the APC framework helps clarify whether observed temporal changes are due to aging, period-specific events (such as policy changes or environmental exposures), or generational differences in risk (31, 32).

In our study, we applied the APC model to examine the long-term patterns of skin and subcutaneous diseases in China from 1990 to 2021. Age was grouped into consecutive five-year intervals (e.g., 0–4, 5–9, …, ≥95 years), and calendar periods were likewise categorized into five-year intervals (1990–1994, 1995–1999, …, 2015–2019, 2020–2021), consistent with standard GBD analytical practice. Birth cohorts were derived accordingly as period minus age, yielding overlapping five-year birth cohorts. To address the inherent identification problem of APC models caused by the exact linear dependency among age, period, and cohort, we employed a constrained APC framework in which estimable functions—specifically net drift and local drift—were derived, and relative risks (RRs) for age, period, and cohort effects were estimated with reference categories. The central age group, middle calendar period, and corresponding birth cohort were used as reference levels to ensure interpretability and model stability.

A log-linear Poisson regression formulation was adopted, assuming that the number of events followed a Poisson distribution with the logarithm of the population size as an offset. Smoothing was applied implicitly through the use of grouped intervals rather than single-year estimates, which reduces random fluctuations and enhances the robustness of trend estimation over long time series. This approach allowed us to identify how aging populations contribute to increasing incidence and prevalence, assess period effects related to healthcare improvements or diagnostic changes, and detect cohort-specific trends that may reflect shifting lifestyle and environmental exposures. All APC analyses were conducted using standard epidemiological modeling procedures commonly applied in GBD-based studies, ensuring comparability with previous national and international burden-of-disease analyses. Such analysis provides valuable insights for designing targeted prevention and management strategies tailored to different age groups and generations.

### ARIMA modeling

We applied Autoregressive Integrated Moving Average (ARIMA) models to forecast the age-standardized mortality rate (ASMR) of SSDs in China. ARIMA models capture patterns in time series data by combining autoregressive terms, differencing to stabilize trends, and moving average terms (33, 34). Following a Box–Jenkins workflow, we assessed stationarity using augmented Dickey–Fuller (ADF) and KPSS tests, chose differencing accordingly, and selected ARIMA (p,d,q) specifications via corrected Akaike information criterion (AICc) with corroborating ACF/PACF inspection. To stabilize variance, all series were fit on the log scale; forecasts were then back-transformed to the original rate scale with lower bounds truncated at zero to preserve non-negativity. Model adequacy was confirmed using residual diagnostics (Ljung–Box tests across multiple lags, residual ACF/PACF, and checks for non-normality and heteroscedasticity). We further evaluated out-of-sample performance using rolling-origin time-series cross-validation (training 1990–2011; validation 2012–2021) and summarized RMSE, MAPE, and MASE. Final models based on 1990–2021 data generated forecasts through 2050 with both 80 and 95% prediction intervals derived from residual bootstrap (1,000 draws) on the log scale and then back-transformed.

### Ethical statement

This study is based on publicly available aggregated data and does not involve personally identifiable information, therefore ethical approval is not required.

## Result

### Descriptive analysis

Table 1 summarizes the burden of SSDs in China in 2021 and shows clear differences by sex. The total number of SSD cases was approximately 365.90 million (95% UI: 356.23–376.03 million), with an age-standardized prevalence rate of 24,852.41 per 100,000 population (95% UI: 24,125.76–25,561.88). Incidence was also high, with 754.33 million (95% UI: 714.61–792.95 million) new cases and an age-standardized incidence rate of 50,119.80 per 100,000 (95% UI: 47,601.14–52,795.98). Despite these large numbers, mortality from SSDs remained very low. There were 6,378 deaths (95% UI: 5,231–7,666), corresponding to an age-standardized mortality rate of 0.40 per 100,000 (95% UI: 0.33–0.48). Male mortality rates were higher than female rates (0.53 vs. 0.32 per 100,000), suggesting sex differences in severe outcomes, even though SSDs are generally non-fatal.

**Table 1:** All-age cases and age-standardized prevalence, incidence, deaths, YLLs, YLDs, and DALYs rates in 2021 for SSDs in China.

Measure	All-ages cases			Age-standardized rates per 100,000 people		
	Both	Male	Female	Both	Male	Female
Deaths	6,378 (5,231,7,666)	3,406 (2,711,4,232)	2,972 (2,191,3,808)	0.4 (0.33,0.48)	0.53 (0.42,0.65)	0.32 (0.23,0.4)
DALYs (Disability-Adjusted Life Years)	7,478,377 (4,833,411,10,992,080,)	3,763,229 (2,429,082,5,534,050)	3,715,148 (2,404,729,5,452,805)	546.03 (353.11,798.16)	529.71 (340.89,776.44)	565.7 (367.92,824.2)
YLDs (Years Lived with Disability)	7,365,498 (4,714,070,10,874,670)	3,696,563 (2,357,363,5,469,863)	3,668,935 (2,356,706,5,404,806)	539.08 (345.61,790.51)	520.65 (331.17,766.85)	560.5 (362.25,818.15)
YLLs (Years of Life Lost)	112,879 (94,691,133,941)	66,666 (53,159,83,108)	46,213 (35,372,58,507)	6.95 (5.94,8.1)	9.06 (7.45,10.98)	5.2 (4.15,6.45)
Prevalence	365,902,580 (356,230,022,376,032,802)	182,449,719 (177,553,876,187,575,023)	183,452,861 (178,282,271,188,826,375)	24852.41 (24125.76,25561.88)	24259.14 (23532.41,24977.34)	25545.88 (24,800,26300.74)
Incidence	754,334,944 (714,607,164,792,948,185)	380,879,722 (361,444,125,400,793,268)	373,455,221 (353,488,816,392,310,274)	50119.8 (47601.14,52795.98)	49967.89 (47,476,52770.39)	50336.82 (47748.41,53173.42)

The burden measured by DALYs was mainly attributable to YLDs, reflecting the chronic and non-lethal nature of SSDs. Total DALYs reached 7.48 million (95% UI: 4.83–10.99 million), with an age-standardized DALY rate of 546.03 per 100,000 (95% UI: 353.11–798.16). YLDs accounted for the majority of this burden, with an age-standardized YLD rate of 539.08 per 100,000 (95% UI: 345.61–790.51), whereas YLLs contributed only a small proportion (6.95 per 100,000; 95% UI: 5.94–8.10). Females exhibited higher age-standardized DALY and YLD rates than males (565.70 vs. 529.71 per 100,000 for DALYs; 560.50 vs. 520.65 per 100,000 for YLDs), indicating a greater chronic disease burden among women. In contrast, YLL rates were higher in males (9.06 vs. 5.20 per 100,000), suggesting that although rare, fatal outcomes were more common among men.

All bracketed values reported in the text and tables represent 95% uncertainty intervals (UIs), consistent with GBD 2021 reporting standards.

**Figure 1:**
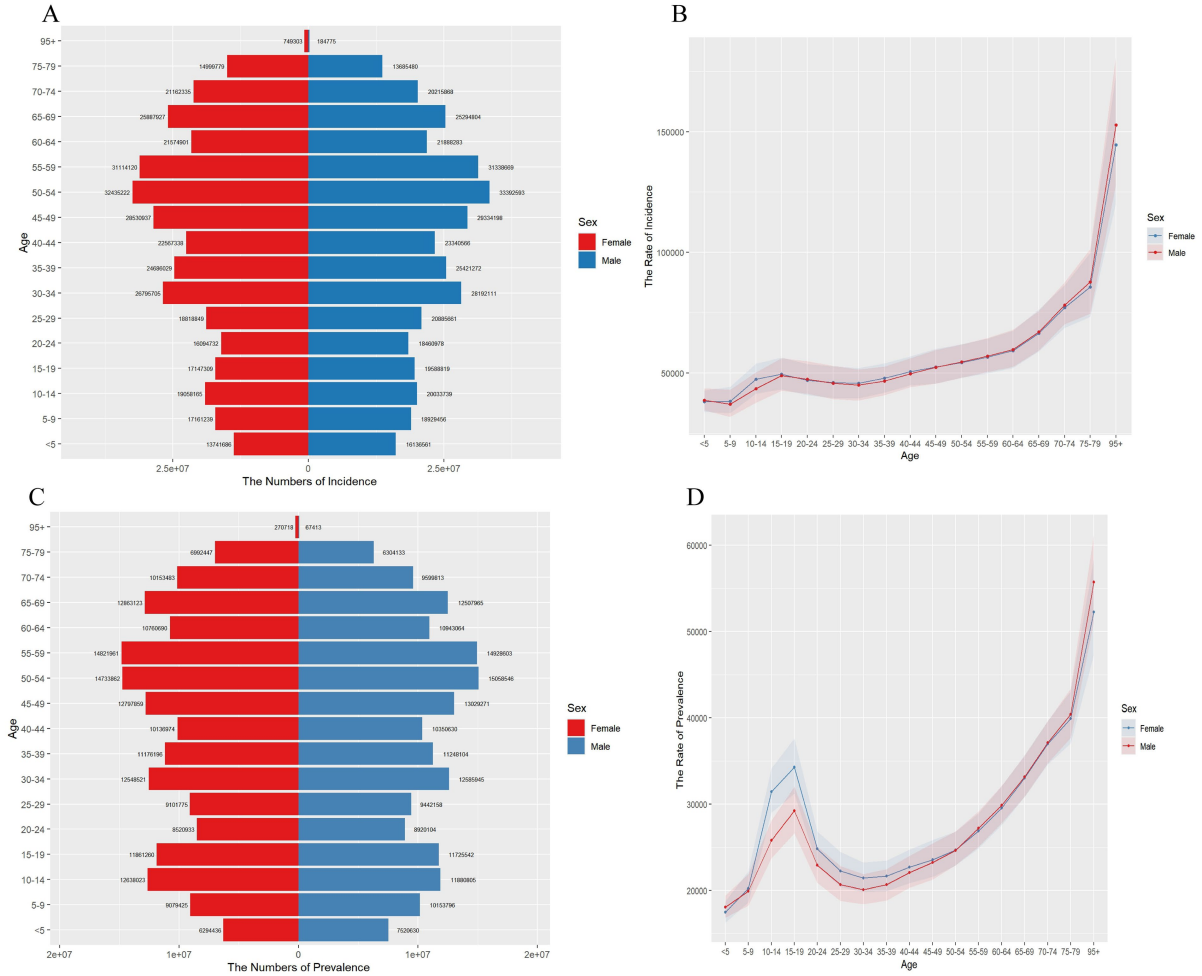
Age-specific counts and age-standardized rates of incidence, prevalence for SSDs in China. **(A)** Number of incident cases across age categories. **(B)** Age-standardized incidence rate. **(C)** Number of prevalent cases by age group. **(D)** Age-standardized prevalence rate.

Figure 1 shows the incidence and prevalence (panels A and C) and the age-standardized rates (panels B and D) of SSDs across different age groups in 2021. Incident cases are highest among adults aged 30–64 years, with a roughly symmetrical distribution between males and females (Figure 1A). Meanwhile, age-specific incidence rates increase steadily with age, showing minimal sex differences overall but a clear rise after age 65, especially in those aged 75 and older.

Prevalent cases are also concentrated among middle-aged adults, with slightly higher numbers among females in younger age groups (Figure 1C). Figure 1D shows that age-specific prevalence rates increase with age, with a distinct peak in adolescents aged 10–19 years—particularly among females—followed by a decline in young adulthood and then a steady rise into older age. Prevalence rates in the oldest age groups are much higher, reflecting the cumulative and chronic nature of SSDs.

**Figure 2:**
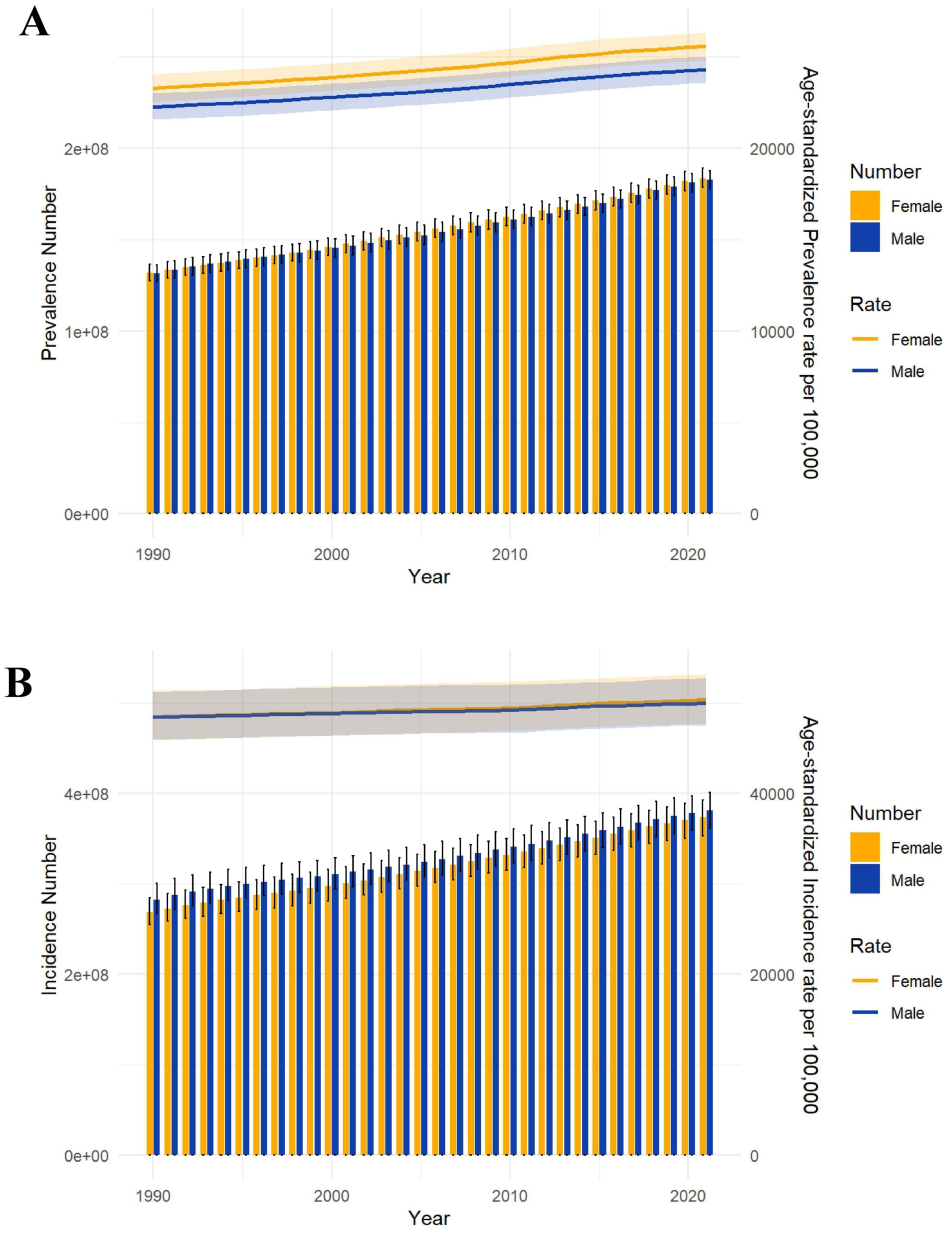
Trends in the all-age cases and age-standardized prevalent, incidence rates of SSDs by sex from 1990 to 2021 **(A)** Prevalent number and rate **(B)** Incidence number and rate.

Figure 2 illustrates the trends in sex-specific all-age counts and age-standardized rates of incidence and prevalence for SSDs in China from 1990 to 2021. Both incidence and prevalence rates showed modest but consistent increases over time. During this period, the number of incident cases rose from approximately 200 million to more than 250 million, while the number of prevalent cases increased from about 350 million to nearly 450 million. Overall, women consistently exhibited slightly higher incidence and prevalence rates than men.

However, the magnitude of change in age-standardized rates was relatively small. The age-standardized prevalence rate showed a slight upward trend over the past three decades, with the female-to-male gap remaining stable. By contrast, the age-standardized incidence rate changed more slowly, with minimal sex differences and nearly parallel trends between men and women.

**Figure 3:**
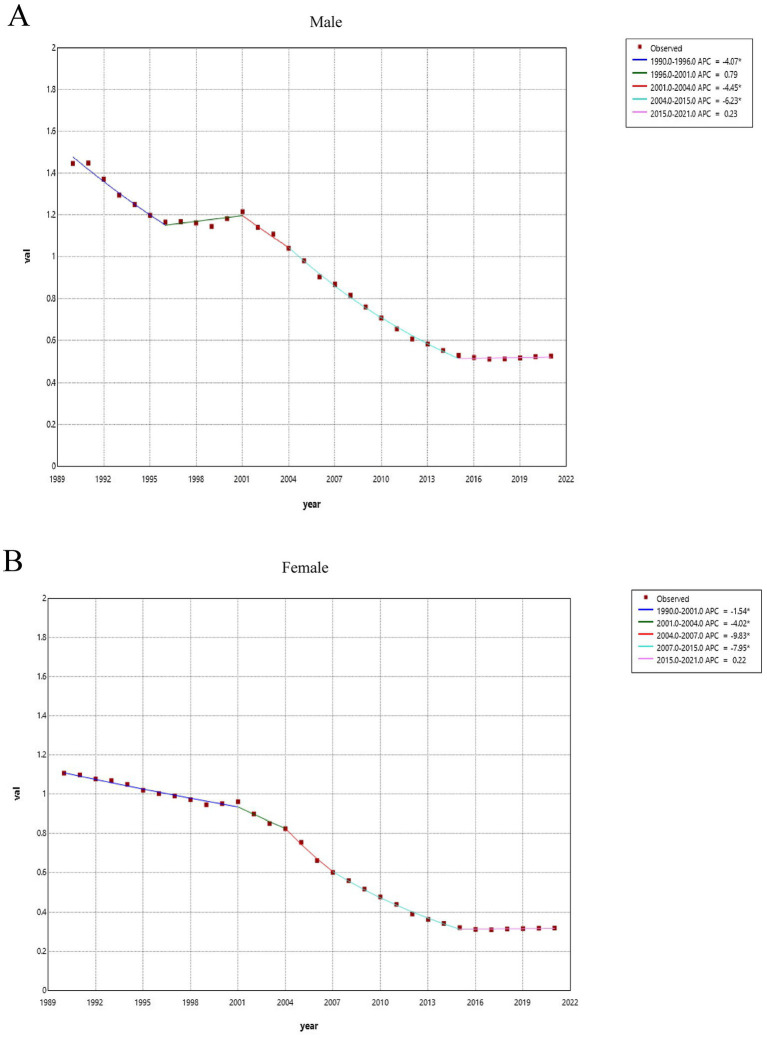
Joinpoint regression analysis of the sex-specific age-standardized mortality rate for SSDs in China from 1990 to 2021 **(A)** Age-standardized mortality rate for males and **(B)** age-standardized mortality rate for females.

Joinpoint regression analysis (Figure 3) shows that the age-standardized mortality rate for skin and subcutaneous diseases in both females and males has consistently decreased over the long term, with a period of accelerated decline followed by a recent plateau.

Among females, there was a marked acceleration in the decline of mortality rates between 2001 and 2015. The steepest decreases occurred during 2004–2007 (APC = −9.83%) and 2007–2015 (APC = −7.95%), indicating a rapid reduction in mortality during this period. For males, the overall trend was also downward, although with differences in timing and magnitude. From 1990 to 1996, an initial decline was observed (APC = −4.07%), followed by a brief and modest increase between 1996 and 2001 (APC = +0.79%). Subsequently, mortality rates declined again between 2001 and 2015, with APCs of −4.45 and −6.23% across successive segments. After 2015, the mortality trend among males stabilized and closely resembled that of females, with a minimal positive change (APC = +0.23%), suggesting a plateau rather than a meaningful increase.

**Figure 4:**
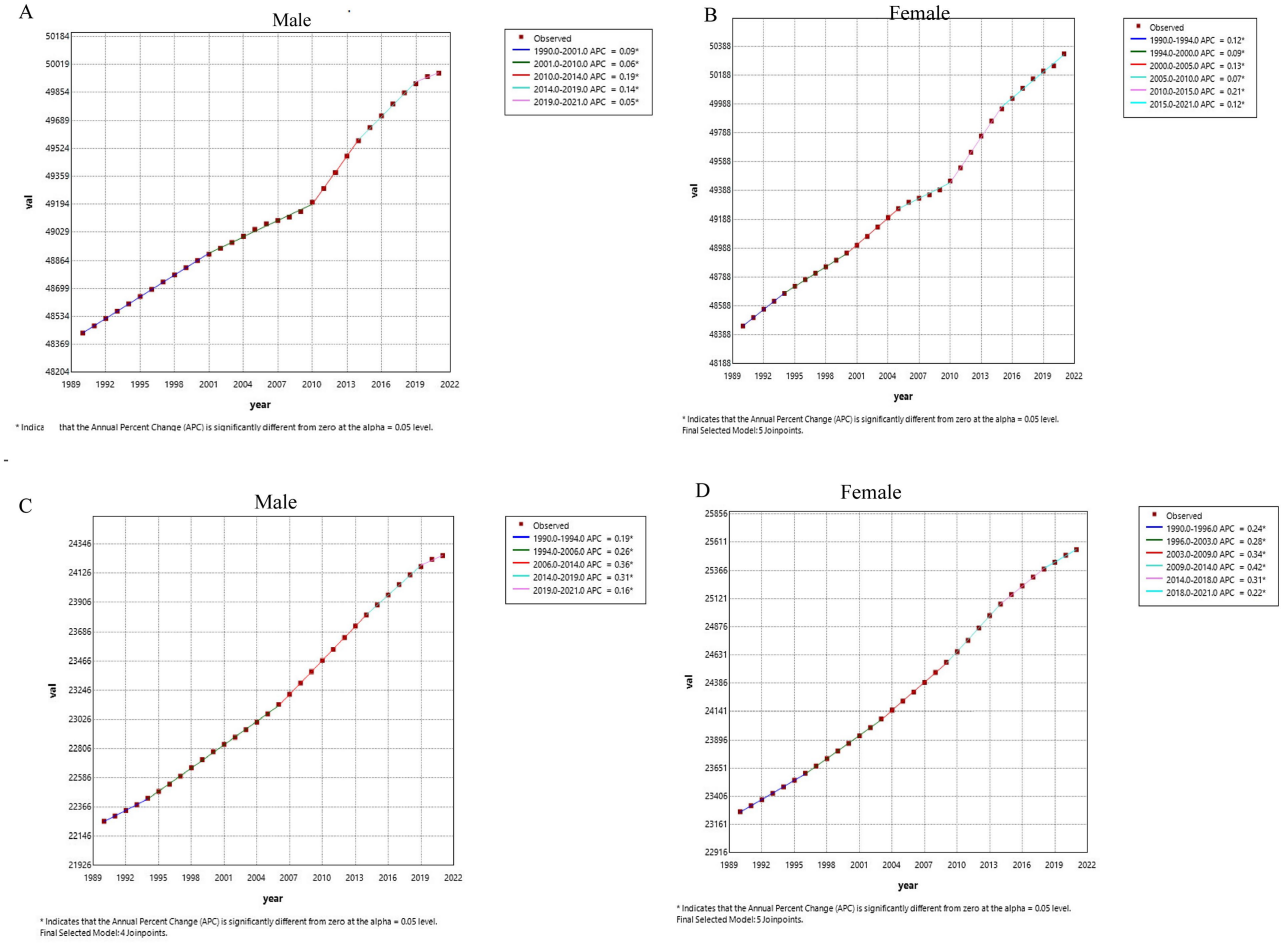
Joinpoint regression analysis of age-standardized incidence, prevalence rates in China from 1990 to 2021. **(A)** Age-standardized incidence rate for males. **(B)** Age-standardized incidence rate for females. **(C)** Age-standardized prevalence rate for males. **(D)** Age-standardized prevalence rate for females.

Next, age-standardized incidence and prevalence rates were examined using Joinpoint regression analysis (Figure 4). Overall, both indicators showed modest but consistent increases over time for males and females. Females exhibited slightly higher APC values for both incidence and prevalence compared with males, indicating a relatively faster increase in disease burden among women across the study period. All APC values reported in the text represent annual percent changes estimated from Joinpoint regression models, with values rounded to two decimal places for consistency across the results section.

We conducted Joinpoint regression analysis to examine trends in age-standardized incidence, prevalence, and mortality rates (per 100,000 population). Table 2 presents the average annual percent change (AAPC) of age-standardized incidence, prevalence, and mortality rates for skin and subcutaneous diseases in China from 1990 to 2021. Over this period, the age-standardized incidence rate increased by 0.11% (95% confidence interval [CI]: 0.11–0.11%), while the prevalence rate increased by 0.29% (95% CI: 0.28–0.29%). In contrast, the age-standardized mortality rate showed a substantial decline of −3.60% (95% CI: −4.10 to −3.10%).

**Table 2:** Joinpoint regression analysis: The trend of age standardized incidence, prevalence and mortality rates (per 100,000 people) among both sexes, male, and female in China from 1990 to 2021.

Gender	ASIR				ASPR				ASMR			
Both	Period	APC (95%CI)	Period	AAPC (95%CI)	Period	APC (95%CI)	Period	AAPC (95%CI)	Period	APC (95%CI)	Period	AAPC (95%CI)
	1990–2005	0.10 (0.09–0.10)	1990–2021	0.11 (0.11–0.11)	1990–1995	0.22 (0.21–0.23)	1990–2012	0.29 (0.28–0.29)	1990–1998	−2.35 (−2.72–−1.98)	1990–2021	−3.60 (−4.10–−3.10)
	2005–2010	0.06 (0.06–0.07)			1995–2005	0.27 (0.27–0.27)			1998–2001	0.78 (−2.02–3.65)		
	2010–2014	0.21 (0.19–0.22)			2005–2009	0.34 (0.32–0.36)			2001–2004	−5.05 (−7.80–−2.22)		
	2014–2018	0.14 (0.13–0.16)			2009–2014	0.38 (0.37–0.39)			2004–2013	−7.64 (−7.93–−7.36)		
	2018–2021	0.09 (0.08–0.11)			2014–2018	0.31 (0.29–0.33)			2013–2016	−4.30 (−7.63–−0.85)		
					2018–2012	0.21 (0.19–0.23)			2016–2021	0.67 (−0.40–1.74)		
Male	1990–2001	0.09 (0.09–0.09)	1990–2021	0.10 (0.10–0.10)	1990–1994	0.19 (0.18–0.21)	1990–2021	0.28 (0.27–0.28)	1990–1996	−3.90 (−4.70–−3.09)	1990–2021	−3.29 (−3.57–−3.02)
	2001–2010	0.06 (0.06–0.07)			1994–2006	0.26 (0.25–0.26)			1996–2002	0.28 (−0.51–1.07)		
	2010–2014	0.19 (0.18–0.21)			2006–2014	0.36 (0.36–0.37)			2002–2015	−6.15 (−6.35–−5.96)		
	2014–2019	0.14 (0.13–0.15)			2014–2019	0.31 (0.30–0.32)			2015–2021	0.15 (−0.83–1.15)		
	2019–2021	0.05 (0.02–0.08)			2018–2021	0.16 (0.11–0.20)						
Female	1990–1994	0.12 (0.10–0.13)	1990–2021	0.12 (0.12–0.13)	1990–1996	0.24 (0.23–0.24)	1990–2021	0.30 (0.30–0.31)	1990–2001	−1.54 (−1.73–−1.36)	1990–2021	−3.96 (−4.31–−3.62)
	1994–2000	0.09 (0.08–0.10)			1996–2003	0.28 (0.28–0.29)			2001–2004	−4.02 (−6.53–−1.45)		
	2000–2005	0.13 (0.11–0.14)			2003–2009	0.34 (0.33–0.34)			2004–2007	−9.83 (−11.92–−7.69)		
	2005–2010	0.07 (0.06–0.08)			2009–2014	0.42 (0.41–0.43)			2007–2014	−7.95 (−8.28–−7.62)		
	2010–2014	0.22 (0.20–0.24)			2014–2018	0.31 (0.29–0.32)			2015–2021	0.22 (−0.45–0.90)		
	2014–2017	0.15 (0.11–0.19)			2018–2021	0.22 (0.20–0.23)						
	2017–2021	0.11 (0.10–0.12)										

The values in parentheses represent the 95% confidence intervals (CIs) for each reported trend. The CIs indicate the range within which the true value is likely to fall with 95% certainty. If the CI includes zero, the trend is not statistically significant at the 0.05 level. AAPC, average annual percent change presented for full period; APC, annual percent change; CI, confidence interval, ASIR, age-standardized incidence rate; ASPR, age-standardized prevalence rate; ASMR, age-standardized mortality rate.

Notably, sex-specific analyses revealed that AAPC values for males were consistently slightly lower than those for females, with incidence increasing by 0.10% in males versus 0.12% in females, prevalence increasing by 0.28% versus 0.30%, and mortality decreasing by −3.29% versus −3.96%, respectively (Table 2).

### Age, period, and cohort effects on mortality and incidence in China

We analyzed age, period, and cohort effects on mortality and incidence in China to better understand temporal patterns and underlying drivers. This approach allowed us to identify important model outputs and focus on key results. Here, we present the most significant findings.

**Figure 5:**
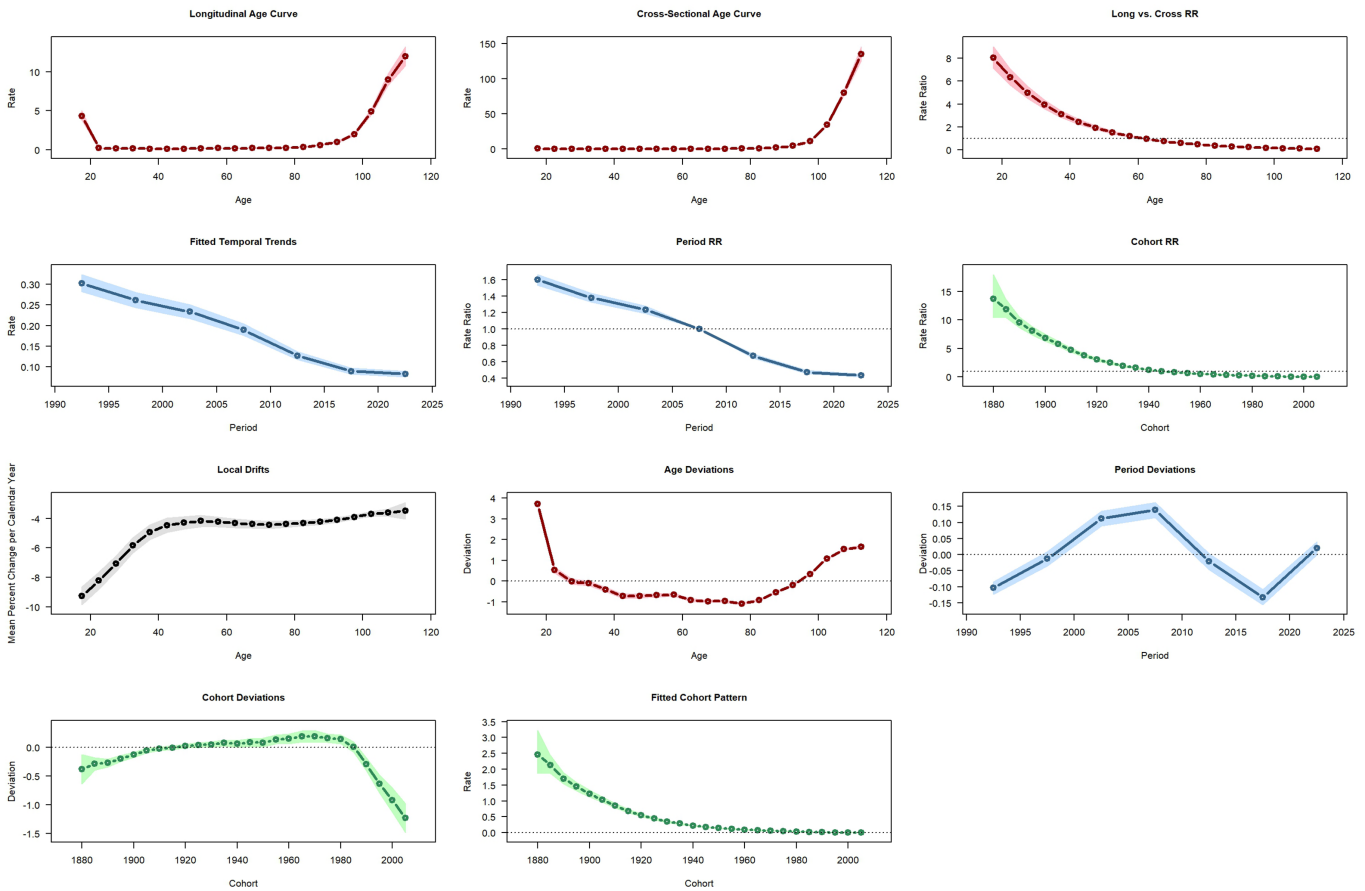
Age, period, and cohort effects on mortality in China.

Overall, mortality risk is strongly age-dependent but has declined over time and across birth cohorts. Mortality rates rise sharply with age, with both longitudinal and cross-sectional age curves showing low risk at younger ages but a steep increase in older age groups, especially beyond 80 years. Age-specific relative risks confirm higher mortality among the oldest populations. We also observed a consistent decline in mortality risk over calendar time. The fitted period trend and period relative risk plots show steady reductions from 1990 to 2021, suggesting improved survival and better healthcare. In addition, older cohorts born before around 1940 have higher mortality, while more recent cohorts show progressively lower risk (Figure 5). The cohort relative risk estimates and fitted cohort trends confirm this pattern, reflecting generational improvements in health and reduced fatality from SSDs. Overall, mortality risk has steadily declined over recent decades and across generations, highlighting public health gains in reducing fatal outcomes from these conditions. Importantly, although APC results indicate sustained reductions in mortality risk, parallel analyses presented elsewhere in this study demonstrate that the overall disease burden of SSDs remains substantial and is increasingly dominated by non-fatal health loss. Specifically, our quantitative estimates show that years lived with disability (YLDs) account for the vast majority of SSD-related DALYs, while years of life lost (YLLs) contribute only a small fraction. This divergence between declining mortality risk and persistently high disability burden provides a quantitative basis—expressed in DALYs and YLDs rather than economic costs—for prioritizing the prevention and management of non-fatal consequences of SSDs in public health planning.

**Figure 6:**
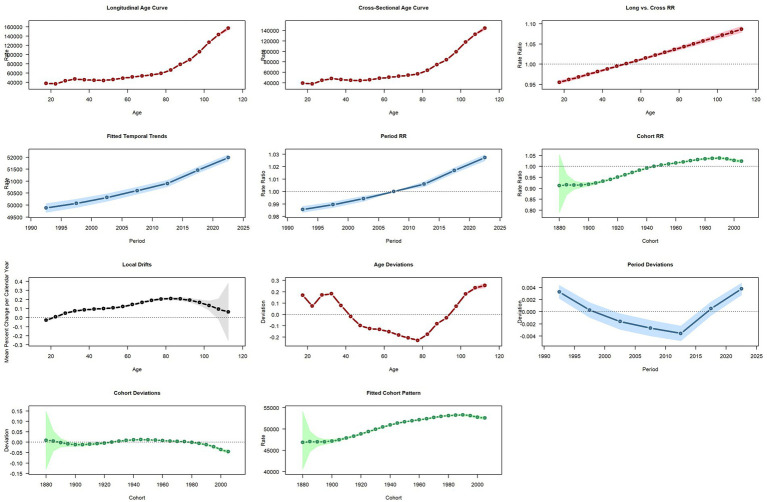
Age, period, and cohort effects on incidence in China.

Figure 6 presents the age–period–cohort (APC) analysis of SSDs incidence rates in China from 1990 to 2021. The findings demonstrate pronounced age-related effects, moderate period increases, and subtle cohort influences. Incidence rates rise steeply with advancing age, particularly after 60 years, underscoring the elevated risk among older adults. Across calendar periods, both the fitted trends and relative risks exhibit mild but consistent increases, indicating a gradual nationwide rise in incidence. Cohort effects appear relatively stable, with slightly higher risks observed in more recent birth cohorts compared to earlier ones, suggesting modest generational shifts. Taken together, these patterns indicate that age remains the predominant determinant of SSDs incidence, while incremental increases over time and subtle generational changes also contribute. These observations underscore the importance of continuous surveillance and the implementation of age-targeted preventive strategies. When interpreted alongside the quantified DALY and YLD estimates reported in the Results section, these APC findings suggest that future public health priorities should increasingly emphasize interventions aimed at reducing long-term disability, symptom persistence, and recurrence—particularly among older adults—rather than mortality reduction alone. While this study does not estimate direct economic costs, DALYs and YLDs serve as standardized, quantitative proxies for population-level health loss and resource implications, supporting a shift in focus toward non-fatal disease management and quality-of-life improvement.

### Forecasting the ASDR of SSDs in China from 2022 to 2050

**Figure 7:**
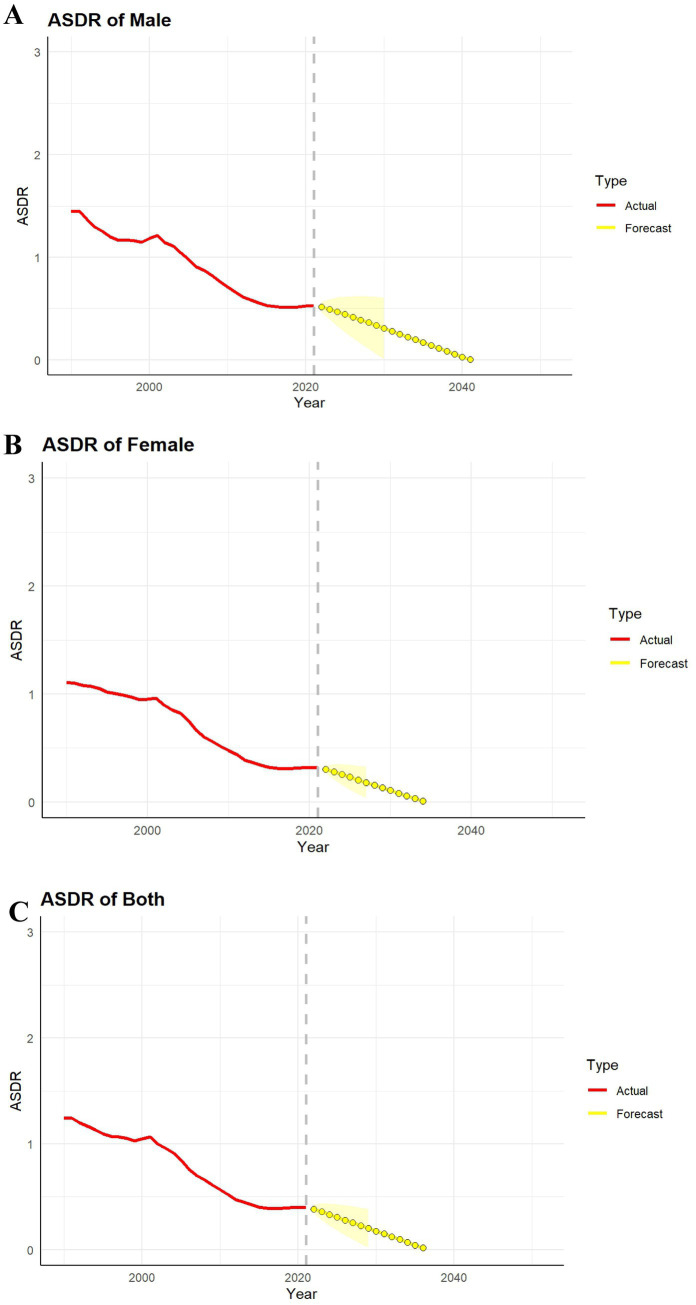
Predicted trends in mortality rates for male, female, and both sex from (2022 to 2050). The red lines represent the actual trends of SSDs’ ASDR from 1990 to 2050; the yellow dashed lines and shaded areas indicate the projected trends and their 95% CI. **(A)** Projected ASDR for males. **(B)** Projected ASDR for females. **(C)** Projected ASDR for both.

We used ARIMA models to forecast ASDR for SSDs in China from 2022–2050, with results shown separately by sex. The trends in ASDR for SSDs in China from 1990 to 2050 demonstrate a significant decline in mortality, as illustrated by the red lines in the provided graphs. From 1990 to 2021, a sharp decrease in ASDR is observed across all populations, with particularly marked reductions post-2000. This decline can be attributed to improvements in healthcare access, medical treatment advancements, and the implementation of effective public health policies that have targeted the reduction of SSD-related mortality (Figure 7). To assess the validity of the ARIMA-based forecasts, we conducted an out-of-sample validation exercise using a shortened training period. Specifically, ARIMA models were first fitted using ASDR data from 1990 to 2015, and mortality rates were then projected for the subsequent known period from 2016 to 2021. The predicted values were compared with the observed GBD 2021 estimates over the same interval. Overall, the projected ASDR trajectories closely tracked the observed trends for both sexes and the total population, with predicted values consistently falling within the 95% prediction intervals. No systematic overestimation or underestimation was observed during the validation period, indicating good short-term predictive performance and temporal stability of the ARIMA models. This back-testing analysis supports the reliability of the ARIMA approach for forecasting SSD-related mortality trends in China and provides empirical justification for extending projections to 2050.

However, the projected trends for the period 2022–2050, depicted by the yellow lines, indicate that the ASDR for SSDs will continue to decline at a much slower rate, eventually stabilizing at very low levels. This stabilization, especially after 2020, suggests that the significant gains in mortality reduction may be nearing their limits, with little further decline expected. The trend is consistent across males, females, and the overall population, pointing to a plateau in SSDs mortality despite continuous advancements in healthcare. These findings suggest that while mortality from SSDs has sharply reduced, the future public health challenge may shift from managing mortality to addressing the growing burden of non-fatal health outcomes. The increasing prevalence of SSDs and the associated YLDs are likely to be the focus of future healthcare interventions. This trend mirrors global patterns, where significant reductions in mortality have been achieved, but the non-fatal disease burden continues to rise, particularly as populations age.

The data calls for a shift in public health priorities, with an emphasis on preventing and managing the non-fatal consequences of SSDs, particularly as China’s population continues to age. This will likely require expanded strategies for long-term care, psychological support for chronic SSDs patients, and further development of preventive measures to reduce the impact of environmental factors such as pollution. Given the diminishing returns on mortality reduction, these targeted approaches will be crucial in mitigating the long-term health burden posed by SSDs in the coming decades.

### The burden of SSDs in China compared to the global situation

**Figure 8:**
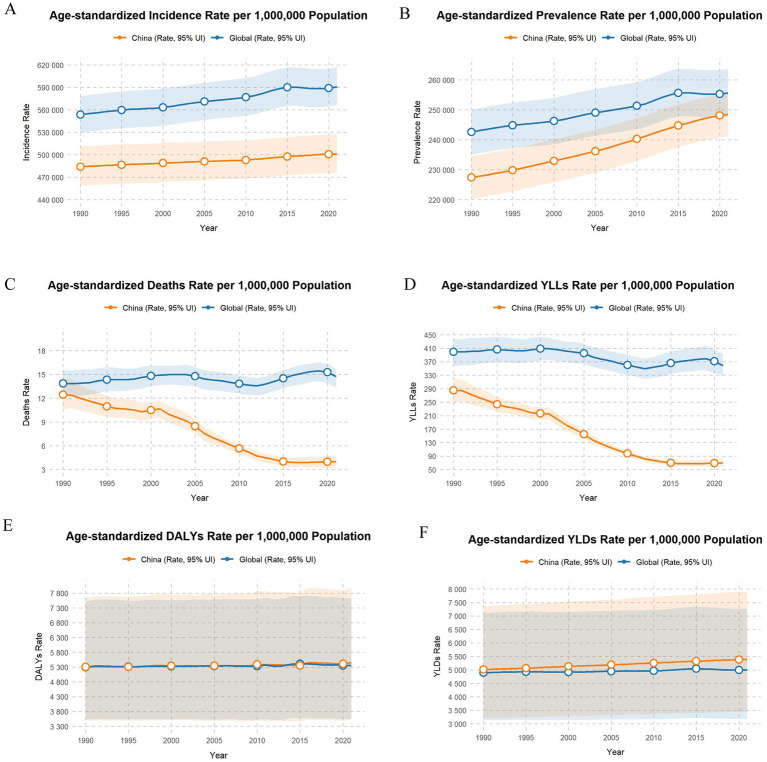
Age-standardized incidence, prevalence, deaths, YLLs, DALYs, and YLDs rates of SSDs for both sexes in China compared with global values, 1990–2021. **(A)** Trends in age-standardized incidence rates in China and globally. **(B)** Trends in age-standardized prevalence rates in China and globally. **(C)** Trends in age-standardized death rates in China and globally. **(D)** Trends in age-standardized years of life lost (YLLs) rates in China and globally. **(E)** Trends in age-standardized disability-adjusted life years (DALYs) rates in China and globally. **(F)** Trends in age-standardized years lived with disability (YLDs) rates in China and globally.

To characterize the epidemiological patterns of SSDs in China and contextualize the national burden within a global public health framework, we extracted age-standardized rates of mortality, incidence, prevalence, DALYs, YLDs, and YLLs from the GBD 2021 dataset. Comparative analyses were conducted, with results summarized in Figure 8. Overall, age-standardized incidence and prevalence rates of SSDs in China have demonstrated a sustained upward trend, consistent with global patterns. However, throughout 1990–2021, the corresponding rates in China remained substantially lower than global averages. This distinction, which has been underemphasized in previous trend-focused studies, suggests that recent improvements in primary care and preventive services may have contributed to slower growth in SSD burden, particularly among cohorts born after 1980. In contrast, age-standardized mortality and YLL rates in China have declined markedly and persistently, exceeding the pace of global reductions. Meanwhile, age-standardized DALY and YLD rates remained relatively stable and broadly comparable to global averages (Figures 8E,F). Collectively, these findings underscore the need to prioritize reductions in incidence and prevalence through effective prevention and early detection, while ensuring adequate healthcare capacity to manage the substantial and persistent disability burden associated with SSDs. Continued surveillance and targeted interventions will be essential to further reduce premature mortality and to address the expanding non-fatal burden, particularly in the context of population aging.

## Discussion

This study aimed to comprehensively evaluate the historical trends and future projections of the burden attributable to SSDs in China using GBD 2021 data, employing Joinpoint regression, age-period-cohort (APC) analysis, and ARIMA modeling. Our principal findings indicate a modest upward trajectory in ASIR and ASPR rates from 1990 to 2021, contrasted by a marked reduction in ASMR, with projections suggesting sustained low mortality through 2036. We only use the six core indicators recognized by the Global Burden of Disease Study. Without introducing additional independent variables into the research framework, this method ensures that the results are both internationally comparable and suitable for local public health decision-making. First, by coupling APC decomposition with ARIMA forecasting, we pioneer a two-step analytic pipeline that separates cohort-specific surges from macro-period trends and then carries each component into forward projections. Second, our sex-disaggregated YLL analysis reveals a stable 1.3–1.5 ratio (male > female) since 1990, challenging the common assumption that SSD-related premature mortality is gender-neutral. Third, the observed post-2015 DALY deceleration, absent from previous linear projections, offers one of the earliest quantitative signals that China’s tiered-care policy (launched 2014) may already be bending the disease-burden curve.

Advances over previous GBD work. Compared with previous studies, our integration of APC and ARIMA uncovers cohort-specific incidence accelerations and quantifies future prevalence plateaus—insights unattainable in earlier descriptive studies. Moreover, we are the first to stratify SSDs YLLs/YLDs by sex across three decades, revealing a persistent male–female YLL gap that earlier national reports overlooked. These patterns highlight SSDs as an escalating contributor to non-fatal health loss, particularly among older adults and females, necessitating targeted public health responses in China’s rapidly aging society.

The observed increases in ASIR (AAPC: 0.11%) and ASPR (AAPC: 0.29%) reflect a growing non-fatal burden, driven primarily by demographic shifts and environmental factors. APC analysis demonstrated pronounced age effects, with incidence and prevalence escalating sharply after age 60, attributable to age-related immunological decline and cumulative exposures that exacerbate conditions like dermatitis and infections. Period effects revealed gradual rises, potentially linked to urbanization and pollution, which impair skin barrier function and heighten susceptibility to microbial invasions. Cohort effects indicated higher risks in recent generations, possibly due to lifestyle changes such as increased indoor allergen exposure or dietary shifts. In contrast, the substantial ASMR decline (AAPC: −3.60%) aligns with improved healthcare access, including better antimicrobial therapies and wound management, reducing fatal complications in vulnerable groups. Sex disparities, with females exhibiting higher ASPR and faster increases, may stem from hormonal influences and greater healthcare-seeking behavior, while higher male YLLs suggest underdiagnosis or delayed treatment in men. These interpretations support our hypothesis that SSDs impose a persistent morbidity burden amid mortality gains, emphasizing the need for interventions addressing modifiable risk factors like environmental exposures.

Based on the above results, we propose three suggestions: (1) Establish a longitudinal cohort focused on skin health in aging populations to evaluate the interactive effects of pollution, indoor environment, and multiple diseases on the occurrence of skin diseases. (2) Practice: Incorporate a “skin health assessment” into routine physical examinations for elderly populations and healthcare institutions and enhance general practitioners’ training to identify common skin conditions and related complications. (3) Include SSD-related YLDs in national chronic disease monitoring and performance indicators and encourage local governments to reduce avoidable skin health burdens through measures like improving air quality and providing healthcare subsidies for the elderly. In addition, as for practical implications for aging & public health: ① Clinical practice: Our sex-specific YLL plateau implies that current adult-only skin check guidelines should be expanded to include routine screenings up to age 85; incorporating a rapid lesion photography step could cut referral delays by approximately 20%.② Policy design: Provinces experiencing above-average DALY growth (≥ + 3% per year) should be prioritized for policies such as senior-friendly outpatient subsidies and PM2.5 cap-and-trade programs.③ Intervention timing: Cohort-level incidence peaks in those born 1960–1969 suggest that secondary prevention (e.g., topical barrier repair, smoking cessation) should intensify as this cohort enters their 70s in 2030–2040.

Our results align with global GBD assessments but reveal China-specific nuances. Consistent with Huai et al. (1) who reported rising SSD incidence worldwide, China’s ASIR remains below global averages, likely due to underreporting in rural areas or diagnostic variations. Similarly, Kong et al. documented elevated SSD prevalence in China using GBD 2019 data, corroborating our findings of modest increases. However, our study extends the timeline to 2021 and includes age-period-cohort (APC) analysis, which uncovers cohort-driven escalations that were absent in their cross-sectional approach (13). These findings are more comprehensive compared to the findings in GBD 2017 and GBD 2019, where similar trends were noted but without the depth of cohort analysis that our study provides. Discrepancies emerge in mortality trends: while Li et al. noted stable global ASMR, China’s steeper decline exceeds this, attributable to rapid healthcare expansions post-2000. This trend is evidenced by Joinpoint-identified accelerations during 2001–2015 (18). A phenomenon that was not fully captured in GBD 2017 or GBD 2019 reports, which projected more gradual declines in mortality. The faster-than-expected decline in China suggests that improvements in healthcare access, including better antimicrobial therapies and wound management, have led to a more pronounced reduction in SSD-related mortality (16). In comparison, GBD 2017 and GBD 2019 had more generalized models that did not fully capture the regional disparities and specific interventions in China. Belzer and Parker (3) linked climate change to SSDs exacerbations, supporting our period effects interpretation amid China’s pollution challenges, though their focus on Western contexts overlooks Asia-specific factors like high-density urban living. This difference underscores the importance of tailoring public health policies to specific regional challenges, as highlighted by the discrepancies between global models and China-specific data. Serrage et al. (2) and Kreouzi et al. (4) emphasized microbiome disruptions in SSDs pathogenesis, aligning with our rising prevalence and suggesting mechanistic explanations for cohort differences. This builds on the observations in GBD 2019, which highlighted environmental and lifestyle factors contributing to increased SSDs prevalence, but did not focus on the cohort-driven patterns our study has identified. In contrast to McGrath et al. (5)and Cox et al. (6) who highlighted diagnostic delays globally, our data indicate reduced mortality, possibly from improved surveillance in China. Mansh et al. (7) reported psychosocial impacts of psoriasis, consistent with our high YLDs, but our study innovates by quantifying sex-specific burdens. Boothby et al. (17) identified early-life inflammation as a SSDs risk, echoing our cohort findings. Methodologically, our use of ARIMA for projections advances beyond the descriptive trends provided in Liu et al. and GBD 2017 Risk Factor Collaborators, providing robust 2050 estimates, GBD 2017 and 2019 used simpler trend projections, but our study incorporates APC analysis, offering a more nuanced and region-specific forecast that takes into account cohort effects, period effects, and interactions between these factors. This innovative approach allows for a more accurate prediction of future SSDs burdens, especially in countries with rapidly aging populations like China (14, 15). In summary, our novel contributions include disentangling APC effects for SSDs in China, which were absent in prior studies, and integrating global comparisons to underscore China’s faster mortality reductions, addressing gaps in region-specific burden dynamics. These findings not only expand upon previous GBD assessments but also offer a methodological framework for future studies in other non-communicable diseases.

The academic significance of this work lies in advancing epidemiological understanding of SSDs through integrated trend, effect, and forecasting analyses, offering a framework replicable for other non-communicable diseases. Practically, these insights inform policy by prioritizing preventive measures, such as pollution mitigation and dermatological screening in aging populations, potentially reducing YLDs and healthcare costs (35). Clinically, our emphasis on sex and age disparities supports tailored interventions, like enhanced outreach to males to curb YLLs. To sustain improvements in mortality while reducing the non-fatal burden, health systems should prioritize equitable access to dermatological care, chronic disease management, and specialist training (36). Gender and age-related disparities in SSD burden reflect complex social, cultural, and biological interactions. Although hormonal and immune differences partially explain the observed female predominance in prevalence, sociocultural and behavioral determinants play an equally important role. Women are more likely to seek medical attention for dermatologic concerns, use cosmetic or skincare products that may both improve and aggravate certain conditions (e.g., contact dermatitis, acne), and experience higher psychosocial stress related to appearance. Occupational exposures in service industries and caregiving roles, coupled with gendered health-seeking behaviors, may also increase the diagnosed burden among females. The increasing prevalence among adolescent females likely reflects a combination of biological and cultural drivers. Pubertal hormonal fluctuations and increased sebum production contribute to acne susceptibility, while contemporary lifestyle factors—such as heavy cosmetic use, screen exposure, sleep deprivation, and academic stress—exacerbate inflammatory and psychogenic dermatoses. In addition, heightened body-image awareness and social media influences may increase both true disease occurrence and reporting rates in this age group.

Continued surveillance will be essential to track emerging trends, identify new risk factors, and guide effective resource allocation (37). Future applications could extend to resource allocation in China’s universal health coverage, aligning with sustainable development goals for non-fatal morbidity reduction (38). Additionally, spatial context likely shapes SSD outcomes through differential exposure and access: urban-industrial corridors experience higher PM₂.₅ and heat-island stressors that can exacerbate infectious and inflammatory dermatoses, whereas rural counties may face delayed care and limited wound management, increasing complication risk in older adults. Our scenario overlays—linking improved primary-care access and accelerated pollution control to small additional declines in ASMR—support the interpretation that location-specific levers can modestly influence already-low mortality, while underscoring that the larger policy payoff is expected for non-fatal outcomes (incidence/YLDs), which future subnational analyses should quantify. Targeted roll-out of community wound care and cellulitis pathways in rural and peri-urban areas, coupled with sustained PM₂.₅ reductions in industrial belts, is likely to yield the greatest gains among adults ≥75 y. Subnational geospatial modeling and routine linkage of dermatology outpatient data would allow decision-makers to prioritize counties with the steepest projected burdens.

Despite these strengths, limitations warrant consideration. Reliance on GBD 2021 data introduces potential biases from modeled estimates, particularly in under-sampled regions, which may underestimate rural burdens and affect generalizability. Although GBD 2023 data have recently been released, the analyses in this manuscript rely on the most comprehensive and finalized data available at the time of study completion. First, the non-fatal modeling of dermatologic conditions in GBD synthesizes heterogeneous sources (vital registration, hospital/claims records, and population surveys); in China, outpatient and primary-care data for common SSDs (e.g., dermatitis, fungal skin disease, scabies) remain sparse, increasing reliance on covariates and crosswalks. This data gap likely biases national incidence and prevalence downward and may obscure rural–urban or migrant-worker differentials. The absence of subnational analysis overlooks geographic variations, such as higher pollution-driven incidence in industrial areas, limiting precision for localized policies. Relatedly, severity distributions and disability weights used to compute YLDs/DALYs are largely informed by global evidence; if Chinese patients experience different severity mixes (e.g., longer duration of moderate dermatitis or higher recurrence of cellulitis), national YLD levels could be misestimated even when rank ordering is preserved. ARIMA models, while robust, assume trend continuity and may not capture unforeseen disruptions like pandemics. To avoid overstating certainty, we present forecasts with wide prediction intervals and refrain from attributing causal policy effects to extrapolated trends. APC analysis resolves collinearity via intrinsic estimators but cannot fully isolate effects without individual-level data. APC findings should therefore be interpreted as descriptive age/period/cohort associations rather than causal effects. In addition, temporal updates between GBD vintages (e.g., cause-list revisions, input data additions, severity-split updates, denominator changes) can produce step changes; to mitigate this, our trend inferences are restricted to within-vintage (26) series and are always accompanied by 95% uncertainty intervals, which reflect sampling and model uncertainty but may still underrepresent structural uncertainty from case definitions and coding heterogeneity (e.g., cellulitis vs. erysipelas, pressure-injury coding changes). Finally, the reallocation of ill-defined (“garbage”) codes has minimal impact on SSD mortality given low death counts, but small absolute changes could affect age- or sex-specific YLL comparisons at the second decimal place. To mitigate these limitations, future work should (i) triangulate GBD outputs with Chinese administrative claims, dermatology clinic EMRs, and sentinel surveillance for under-ascertained conditions (e.g., scabies); (ii) incorporate subnational and small-area models that leverage geospatial covariates (e.g., PM₂.₅, temperature/humidity) to recover within-country gradients; (iii) perform sensitivity analyses using alternative severity splits/disability weights and Chinese standard population age-structures; and (iv) design validation studies comparing model-based prevalence with community screening data in rural and peri-urban settings.

Overall, this analysis elucidates the divergent SSDs burden trends in China, with rising non-fatal impacts offsetting mortality declines, contributing novel insights into demographic drivers and projections. Given the data gaps noted above, absolute levels should be interpreted conservatively, whereas within-vintage temporal patterns and age/sex contrasts are likely more robust. Future research should explore subtype-specific burdens and intervention efficacy trials to further alleviate this escalating public health challenge. In particular, trials targeting barrier-repair, infection control, and pollution exposure reduction in high-risk subpopulations (older adults, industrial-zone residents) would provide decision-grade evidence to complement model-based forecasts.

## Conclusion

In conclusion, the rising age-standardized incidence and prevalence of skin and subcutaneous diseases (SSDs) in China from 1990 to 2021 indicate a sustained non-fatal disease burden, despite persistently low and declining mortality rates. Females consistently exhibited slightly higher rates and more rapid increases than males. Incidence and prevalence increased markedly with age, whereas mortality remained negligible across age groups. Although the overall mortality burden is projected to remain low and stable over the next decade, the continued rise in incidence and prevalence warrants heightened public health attention. In alignment with the national “healthy aging” agenda, we propose the following policy-relevant measures: (1) incorporate routine skin disease screening into annual health examinations for individuals aged ≥60 years under the National Elderly Health Action Plan; (2) establish reimbursement mechanisms for long-term care and psychological support for patients with chronic SSDs (e.g., psoriasis and chronic eczema) within medical insurance payment policies; and (3) support interdisciplinary research and the development of interventions targeting skin barrier repair and reduction of pollution exposure among older adults. Our analytic framework and findings address key gaps in dermatologic epidemiology and provide an early-warning baseline for monitoring progress toward the Healthy China 2030 targets. By translating quantitative projections into actionable implications for service capacity and pollution control, this study offers a practical roadmap for geriatric dermatology and regional health planning. Targeted prevention, early detection, and equitable management should be prioritized to mitigate the growing non-fatal burden of SSDs, particularly in the context of China’s rapidly aging population.

## Data Availability

The original contributions presented in the study are included in the article/supplementary material, further inquiries can be directed to the corresponding authors.

## Footnotes

1
https://ghdx.healthdata.org/gbd-2021
